# Effects of Game Mode in Multiplayer Video Games on Intergenerational Social Interaction: Randomized Field Study

**DOI:** 10.2196/29179

**Published:** 2022-02-16

**Authors:** Carmen Zahn, David Leisner, Mario Niederhauser, Anna-Lena Roos, Tabea Iseli, Marco Soldati

**Affiliations:** 1 School of Applied Psychology University of Applied Sciences and Arts Northwestern Switzerland Olten Switzerland; 2 School of Engineering University of Applied Sciences and Arts Northwestern Switzerland Windisch Switzerland

**Keywords:** video games, computer games, older adults, game mode, serious game, social interaction, video analysis methods

## Abstract

**Background:**

Maintaining social relationships is a basic human need and particularly essential in old age, including when living in a retirement home. Multiplayer video games can promote positive social interactions among players from different generations while playing. Yet, such facilitation of positive social interactions depends on specific game design. To systematically investigate the effects of game design on social interaction between seniors and their coplayers, the game *Myosotis FoodPlanet* was developed in this study, and the impacts of 3 different game modes on social interaction were compared in a controlled field trial.

**Objective:**

This study aims to compare the effects of 3 different game modes (competitive, cooperative, and creative) on social interactions (verbal and nonverbal communication) between seniors and their younger coplayers.

**Methods:**

This study was conducted in a Swiss retirement home as a controlled field trial. Participants were residents of the retirement home (N=10; mean age 84.8 years, SD 5.9 years) and played in pairs with their caregivers. Each pair played 3 game modes in random order. This resulted in 30 game sequences of 20 minutes each. A within-subject design was applied with *game mode* as the within-factor and *social interaction* as the outcome variable. To assess the quality of social interaction, 30 video-recorded game sequences were analyzed based on an event sampling method.

**Results:**

Analysis of variance for repeated measurements revealed significant effects: there was significantly more verbal communication in the creative mode than in the cooperative mode (*P*=.04) with a strong effect size (Cohen *f*=0.611). An examination of verbal communication revealed more *game-related* communication in the creative mode than in the cooperative mode (*P*=.01) and the competitive mode (*P*=.09) with marginally significant effects and strong effect sizes (Cohen *f*=0.841). In addition, significantly more biography-related communication occurred in the creative mode than in the cooperative mode (*P*=.03), with a strong effect size (*r*=0.707). Regarding nonverbal communication (eg, laughing together), analysis of variance for repeated measurements showed significant differences among the game modes (*P*=.02) with a strong effect size (Cohen *f*=0.758). Results showed that there was significantly more laughing together in the competitive mode (competitive>cooperative>creative).

**Conclusions:**

The results show that game mode can be an important factor for shaping the social interactions of players playing together. Compared with other modes, creative game modes can increase verbal communication. In contrast, competitive modes may stimulate more laughing together. This has important implications for game design and the use of computer games to promote social interaction between seniors and their coplayers in practice.

## Introduction

### Playing Digital Games as Social Activity Connecting People From Different Age Groups and Generations

Maintaining social relationships is a basic human need and therefore is highly relevant to psychological well-being [1]. This is particularly true in old age [2-5], including when living in a retirement home. Multiplayer video games can promote well-being and the maintenance of social relationships [6-8] because they can facilitate *positive* social interactions during play, even for players from different generations [9-14]. Imagine, for instance, a visiting grandchild playing a video game with their grandparent in a retirement home, or imagine care persons and residents playing together during activation therapy or recreation in a common room. Playing together is a rewarding experience [15] that connects people through joint action, cooperation, or playful and unthreatening competition. In this way, it can link people from *different* generations, bridging the gap among them. Research indicates that social interaction and entertainment are among the main motivating factors for older adults to play video games [16-18]. Specifically, players enjoy interacting with others, watching others playing, and talking about the game [19-21]. Digital technology, which is ubiquitous in the lives of younger and adult people, has been investigated as a tool to connect generations, including those of advanced age [2]. For instance, intergenerational digital games can enhance social bonding [22] and pave the way for improved communication among players [23]. The term *intergenerational*, thereby does not only include family bonds (eg, grandchildren and grandparents) but can also be considered in a wider sense in terms of age (eg, older and younger people) or community life (eg, youth and older adults) [24].

Taken together, playing video games seems to be a meaningful way to foster positive intergenerational social interaction between younger and older people, which, in turn, is likely to improve psychological well-being in the long run. As emphasized in a review [25] and various studies [26,27], games can have positive effects on the physical, cognitive, social, and emotional states, especially in older adults.

However, there are important research gaps that must be considered concerning the potential of video games in real-life scenarios, especially those concerning specific game design decisions and their impact on social interactions. For example, de la Hera et al [23] state: “The decision to engage players in collaborative, competitive or cooperative competitive games has relevant implications on the effects of these practices.” Previous research on intergenerational digital games describing major research gaps recommend that future empirical studies should directly compare different forms of playing to discover the effects of intergenerational interactions [24]. Likewise, recent research related to digital games in neurorehabilitation has addressed similar research gaps concerning different game modes [28]. Therefore, it is of utmost importance to further contribute to the scientific knowledge on the effects of game design decisions on players’ behaviors via systematic empirical studies. In addition, it has been recommended by previous research on intergenerational games that future research should include more types than only grandparent–grandchildren interactions [23].

In our study, we investigate the possible effects of specific game design decisions regarding game modes on social interactions among players from different age groups in a retirement home. In the following section, we present the rationale behind our game design decisions.

### Game Design Decisions and Possible Influences on Players’ Social Interactions

In multiplayer game design [29], *social game mechanics* are used to initialize and increase the social interaction among players within the game. Systematic empirical research is rare in this area; however, isolated evidence has demonstrated that design for social interaction can impact players’ behaviors in old age—not only concerning their verbal behavior but also their nonverbal behavior; a study comparing social interaction design of a pervasive game (pervasive game here interrelates to the close connection between a web-based game and the physical world), as opposed to *no* social interaction design, revealed significant and positive effects on promoting physical activity in older adult players [30].

More specifically, social game mechanics provide different configurations—*game modes* that can influence social interaction among players. The established modes are (1) competitive, if 2 players compete against each other, whereby only one of them can win [31] and (2) cooperative, if 2 players *operate together*, having a dedicated task each, but winning the game together. These contrasting modes are both able to stimulate social interaction and motivation to play [32-34]. They correspond to basic categories of human social interaction behaviors, which are well-known from the long history of social psychology research [35]. In addition, modern games provide a (3) *creative* mode in which the game does not imply any rules and the players are free to explore or modify the game world in a creative way. Such games are referred to as open-ended simulation games or sandbox games [36,37].

Existing research reveals an ongoing debate regarding the influence of game modes on social interaction. Although some [35] assume an increased willingness to communicate in cooperative game structures, others [38] argue for the existence of a correlation between competitive game structures and social interaction. More detailed findings and theoretical considerations on the different game modes and their influence on social interaction are presented in the following section.

### Game Modes and Players’ Social Interactions—Empirical Evidence

#### Competitive Mode and Social Interaction

Research on game gratification shows a positive relationship between social and competitive motives [39,40], and players who engage with games primarily because they seek social interaction are often also competitive gamers [38]. This correlation can be explained by the basic human need for control, according to the fundamental interpersonal relationship orientation theory [41]: competition with others or trying to control each other is an essential part of interpersonal dynamics [38]. Nevertheless, in contrast to younger players, older players find competition in playing a minor motivator [16,18,22,27], unless there is indirect competition against other teams [42]. In addition, older players have been found to largely reject reflex-oriented games such as fighting or racing games. They experience such games as more difficult, less interesting, and hence less enjoyable to play, owing to their age-related physical condition or disabilities [43].

#### Cooperative Mode and Social Interaction

Empirical findings suggest that cooperative video games support positive interdependence (eg, for video games) [44,45], meaning players need each other to fulfill a certain task and all members must contribute their knowledge and skills for the group or team to be successful [35]. Positive interdependence plays a crucial role in improving intergenerational social interactions [23]. Thus, cooperative game structures should likely lead to increased willingness to communicate as a team, facilitating the exchange of important information, sharing of ideas, and reacting to the ideas shared by others [35]. However, to the authors’ knowledge, there have been no clear empirical results confirming this assumption for older adults or intergenerational games.

#### Creative Mode and Social Interaction

The creative game experience has been seen to increase when the game does not specify any right or wrong paths [36]. This can foster creativity because players collaboratively generate new ideas beyond what they could have come up with on their own [46-48]. Following this, the given level of freedom in the creative game is likely to initiate and increase communication (about game-related activities and thoughts) and social interaction (joint decision-making) among players. Talking about new ideas or things that are out of the ordinary can lead to discussion of new topics and even more communication. This, in turn, should further increase social interactions. Empirical studies examining this relationship are lacking.

Taken together, these empirical findings suggest that all 3 modes can stimulate social interaction and motivation to play [31,33]. However, empirical studies comparing different modes in relation to social interaction are inconsistent (competitive vs cooperative modes) or lacking (creative mode). As the theoretical considerations described above suggest that there may be differences, a comparative analysis investigating the differential effects of cooperative, competitive, and creative game modes on intergenerational social interaction can fill the research gap as described previously [23] and inform game designers on how to design games that stimulate intergenerational social interaction [22].

### Goals of the Field Trial

The goal of this study is to provide original results that contribute to improved scientific knowledge about the effects of social game mechanics (game modes) on social interactions between older players and younger coplayers. To accomplish this, a controlled field trial was presented to investigate these impacts with older participants from a retirement home. The game used for the trial is a serious multiplayer game called *Myosotis FoodPlanet* (see the *Materials and Tools* section and Multimedia Appendix 1) designed specifically as an intergenerational game for use in retirement homes.

This study aims to compare the impacts of 3 different game modes (competitive, cooperative, and creative) on social interactions between seniors and their younger coplayers during game play. With reference to the theory described above and previous research, we assumed that the game modes would differ in the extent to which they influence social interaction. Therefore, we differentiated between verbal social interaction (H1) and nonverbal social interaction (H2) among players. Specifically concerning H1, we expected the creative game mode to stimulate the highest amount of verbal social interaction, as explained earlier. Despite somewhat controversial findings regarding the competitive and cooperative modes, we further assumed that a cooperative mode would stimulate more verbal social interaction, compared with the competitive mode, based on related research [35]. In brief: Creative Mode>Cooperative Mode>Competitive Mode.

Regarding nonverbal social interaction (H2), we hypothesized that there would be differences among game modes; however, owing to the extremely limited research on nonverbal interaction in video games, the assumptions were nondirectional, and our research remains explorative. The study was conducted before the COVID-19 pandemic, when physical interactions (hand shaking and touching another’s hands, shoulders, etc) were still not restricted owing to potential health risks.

## Methods

### Participants and Study Design

In all, 10 older residents from a Swiss retirement home voluntarily participated in a randomized controlled field trial during their leisure time (7 women and 3 men; mean age 84.8 years, SD 5.85 years; range 76-93). The older participants were healthy, with the exception of minor age-related impairments (ie, minor physical limitations and no severe dementia). A within-subject study design was applied with 3 game modes (competitive, cooperative, and creative) administered in a randomized order. This resulted in 30 game sequences. In all, 4 care professionals, 3 activation therapists and 1 nurse (4 women; mean age 44, SD 15.60 years; range 21-55 years) participated as coplayers of a younger generation to ensure the residents’ safety at all times during study participation and liability to ethical standards. The study was not part of any therapeutic program and did not include any clinical interventions. Social interaction was the only outcome variable (for details on measures, see the *Measures of Social Interaction* section). No health-related outcomes were addressed in the study. According to the Swiss Federal Human Research Act, the study was not liable for registration.

### Materials and Tools

The game used in this study is a serious multiplayer game called *Myosotis FoodPlanet*, which was designed specifically as an intergenerational game for use in retirement homes. It is a game involving cooking Swiss cheese fondue together, which is a traditional and well-known dish in Switzerland. From reminiscence therapy [49], it is known that food is an ideal topic for stimulating social interaction [50], because anybody can be assumed to have an opinion on food, and the interests of different generations can easily be taken into account [11]. *Myosotis FoodPlanet* was developed in the multidisciplinary research project Myosotis Garden [37]. The games developed in this project were designed to provide entertaining positive activity, thereby triggering intergenerational communication and enabling players to find new and exciting access to the memories and biographies of older people [23].

**Figure 1:**
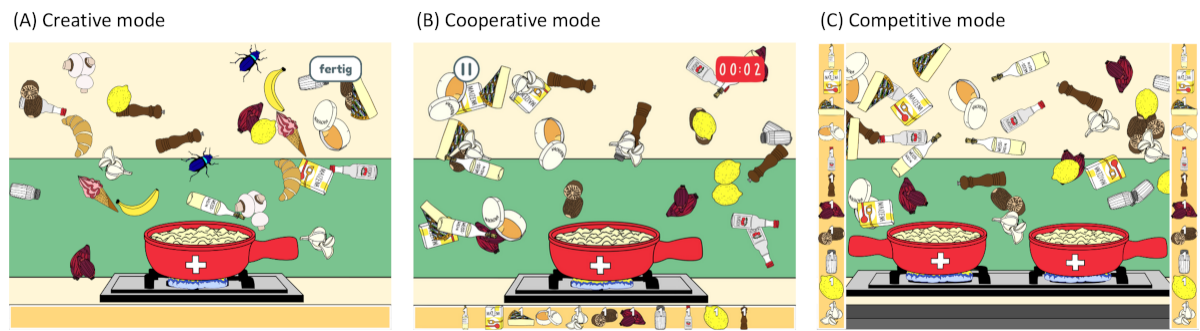
Game modes: (A) creative, (B) cooperative, and (C) competitive.

In *Myosotis FoodPlanet*, two players—one older person and one younger care professional in the case of this study—jointly prepare a Swiss cheese fondue on an iPad Pro (32.8 cm) by dragging floating ingredients into a fondue pot (Figure 1). To ensure that older adults, despite possible age-related handicaps, recognize the ingredients, a computer-generated voice announces the name of each ingredient when it is tapped. In addition, the names of the ingredients are presented in a written form. A traditional Swiss folk melody plays in the background, contributing to the creation of a pleasant atmosphere. The game is designed for 2 players sitting in the same room and sharing the touch screen. The entire screen is used by both players, as opposed to a split-screen mode. Although the game is commonly played synchronously, the creative mode also allows a turn-based approach, where one after the other, the players add ingredients to the pot. A total of 3 variants of *Myosotis FoodPlanet*, each offering a different game mode (Figures 1A-C) were used in our field trial.

In the *competitive mode* (Figure 1A), each player prepares a given fondue recipe as quickly as possible. Each player has their own fondue pot and their own but identical given ingredient list that is displayed on the respective side of the screen closest to the player. The goal of the player is to drag the given ingredients into their own pot faster than the other player. It is also possible to drag the ingredients needed by the opponent into one’s own pot. The player who collects all the listed ingredients first wins.

In the *cooperative mode* (Figure 1B), players work together to prepare a fondue by collecting ingredients from a list of typical fondue as quickly as possible into one pot for both. The ingredients are listed in the bar at the bottom of the screen. The order in which the ingredients are collected is irrelevant, and the ingredients can be dragged repeatedly. Once all the listed ingredients have been dragged into the pot, the total time taken and the current high score (best time of all players) are displayed.

In the *creative mode* (Figure 1C), players collaboratively and freely choose ingredients for their cheese fondue. Each ingredient can be dragged into the pot as often as desired, while the bar at the bottom of the screen shows the ingredients already added. Players can choose typical (eg, Gruyère cheese and white wine) as well as atypical (eg, bug and soft ice) fondue ingredients. The latter will likely increase the fun factor and consequently the social interaction. When an atypical ingredient is added, a colored splash appears on the screen, accompanied by a squeaky sound effect. After a short period, the splash disappears, but the cheese fondue now is colored similar to the atypical ingredient (eg, adding a bug turns the fondue blue). The players end the game manually. As a reward, the players obtain the prepared fondue in a recipe form. In addition, the system automatically names the recipe with humorous names (eg, *Dancing Hans* or *Singing Theodora*) to increase the fun factor and social interaction.

### Procedure

**Figure 2:**
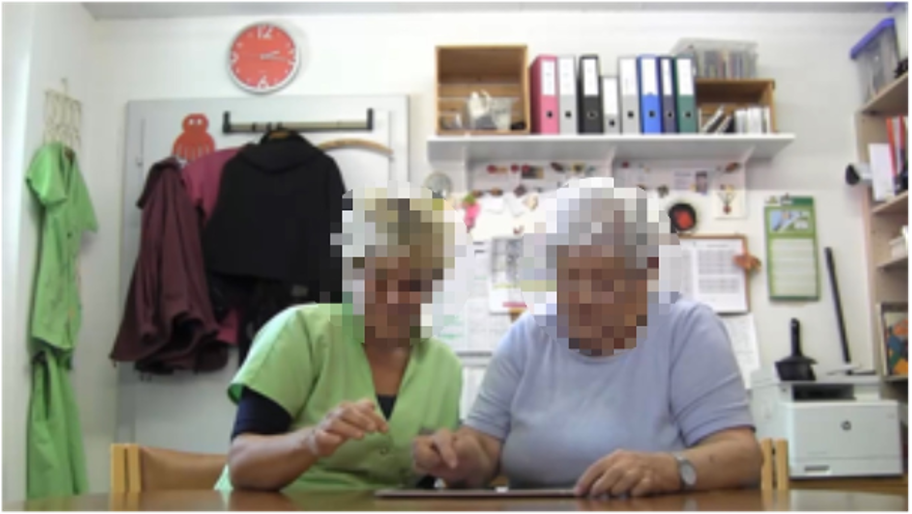
Study setting: game sequence.

The study was conducted in the activation room (a room with which the participants were familiar) of the retirement home as a free-choice afternoon leisure activity. Each player participated on 3 different days and played 1 of the 3 game modes in random order. Each game sequence lasted for a maximum of 20 minutes. Participation was voluntary, and it was made clear that participants could withdraw their participation at any time without any consequence. The participants were informed beforehand about the goals of the research, duration and procedure of the study, voluntary nature of participation, and protection of their data. Written informed consent was obtained from all test participants before starting each game sequence, as well as from the institution before starting the trial. The players were seated next to each other so that their dominant hand could easily access the tablet computer (Figure 2).

This procedure was performed in accordance with the Helsinki Declaration of 1975, as revised in 2000. As noted, it was not liable to registration according to the Swiss Federal Human Research Act.

### Measures of Social Interaction

Social interaction was measured by verbal indicators, such as verbal communication and nonverbal indicators, such as laughing together or eye contact [51]. Thus, data gathering was based on video recordings of verbal and nonverbal communication among players. In all, 30 game sequences were recorded using 2 video cameras. Gameplay recordings, as well as observations, rank among the most frequently used methods to investigate the possible influences of digital game playing on social interaction [23]. Video analysis has proven to be a suitable research tool for observing behavior and social interactions [52]. It enables the storage and reproducibility of complex social interaction data, allowing for multiple observations by different observers at different times, depending on the individual research questions of interest. It was initially intended that self-assessment questionnaires with questions about the players’ subjective experiences and well-being would also be administered; however, some older participants had difficulty understanding the questions. (It was unclear whether this was owing to their Swiss language, minor impairments, or a lack of motivation to fill out the questionnaires.) Owing to this, the self-assessment questionnaire component was removed.

### Data Analysis

**Figure 3:**
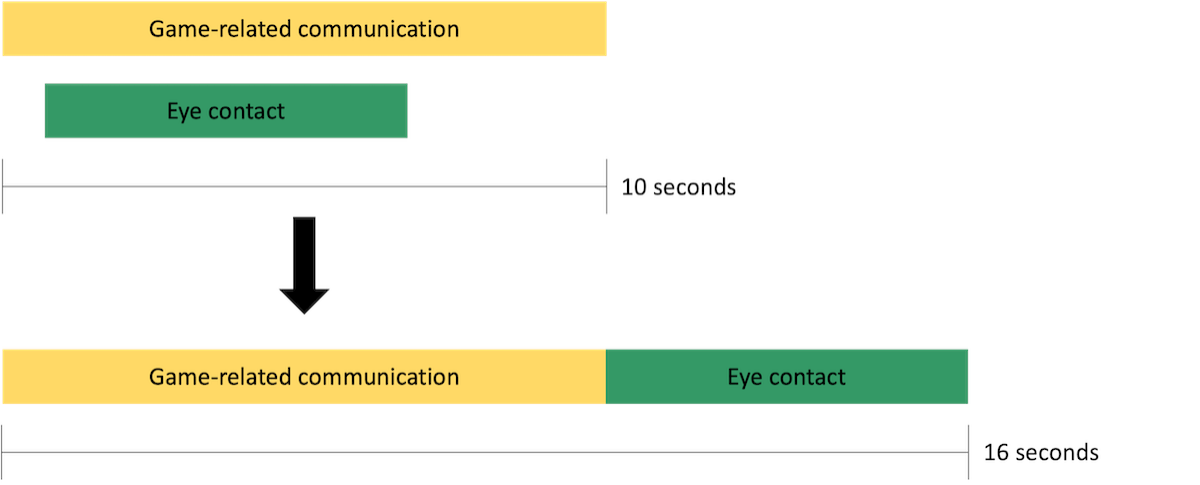
Coding example—overlaps.

The video data gathered comprised 30 recorded game sequences of approximately 8 hours (7 hours 53 minutes 25 seconds). For video data analysis, a 2-step *coding and counting* approach [53,54] was applied. In the first step, the 30 recorded game sequences were coded using the text analysis software MAXQDA 2018 (VERBI GmbH, Berlin, Germany) based on a preliminary category system (*event sampling* [55]). In the second step, the final category system was developed, including new categories emerging from the data, by rewatching video recordings in an iterative process. As a result, social interaction in terms of *verbal* communication among players was divided into categories: *game-related communication, fondue-related biography, general biography, help seeking,* and *help giving*. Social interaction in terms of nonverbal communication was divided into *laughing together, eye contact,* and *body contact*. Table 1 lists the definitions and corresponding anchor examples [56] for each category. The communication behaviors were then systematically coded by 2 trained raters (duration of behavior in minutes and seconds; mm:ss). The raters were trained on examples from the videos; the raters discussed these examples and resolved any uncertainties. They then coded the videos. Intercoder reliability among the raters resulted in substantial agreement (*к*=0.69). Finally, the durations of all relevant behavior indicators of a given code were summed up. Verbal communication (eg, game-related communication) and nonverbal communication (eg, eye contact) occurred simultaneously. Owing to overlapping codes caused by this occurrence of simultaneous communication, finally, as an artifact—the sum duration of each observed communication exceeds the effective observation time of total social interaction (Figure 3).

**Table 1:** Category system for the observation of social interaction behaviors.

Category			Definition		Anchor examples^a^
**Verbal communication**					
	Game-related communication	All statements resulting directly from the game (eg, discussing results, outcomes, or new strategies)		“Oh my God, that’s fun” and “You won again, congrats”	
	Fondue-related biography	When players discuss the method of preparation or consumption of a cheese fondue in the past (ie, anecdotes)		“My son-in-law doesn’t like garlic. So, when he came to visit, I always had to make a cheese fondue without garlic” and “Do you like onions in the fondue?”	
	General biography	When personal biographic information is shared (when players tell or ask about their professional life, family, military, childhood, etc)		“You know, I used to work in a cheese factory and there was this one customer, who...” and “Originally I am from Austria, but after the war my family moved to Switzerland”	
	Help seeking	When older participants actively ask for help for technical operation of the game or when actively asking about content aspects during the game		“Why can I not grab that cheese over here?” and “Have I collected all the needed ingredients?”	
	Help giving	When coplayers answer questions regarding technical or content matters. Furthermore, when coplayers help the older adults by giving them hints		“Try using your fingertip instead of your fingernail” and “Look, over there is the onion you still need to collect”	
**Nonverbal communication**					
	Laughing together	When players laugh out loud together; also includes when one person laughs and simultaneously talks and the other smiles		N/A^b^	
	Eye contact	When players look directly into each other’s eyes (ie, eyes are meeting)		N/A	
	Body contact	When one player is patting the other on the back, or when one player is touching the other’s hand or arm		N/A	

^a^Anchor examples are verbal statements translated from Swiss German.

^b^N/A: not applicable.

Results were visualized using MAXQDA 2018, including the overlapping of codes. Thus, it was possible to illustrate which verbal and nonverbal categories tend to occur simultaneously (Table 2). For quantitative analyses, data were transferred to the statistical software SPSS. Owing to the small sample size, the data were checked for normal distribution and variance homogeneity. Statistical analyses compared each category of social interaction by using absolute values (ie, mean duration of social interaction in minutes and seconds; mm:ss), as well as relative values, (ie, percent duration in relation to the total playing time) for the 3 game modes. Hypotheses were tested by analyses of variance with repeated measurements or its nonparametric counterpart (Friedman test).

**Table 2:** Simultaneous occurrence of verbal and nonverbal codes.

	Laughing together, n	Eye contact, n	Body contact, n
Game-related communication	604	556	83
Fondue-related biography	31	204	1
General biography	12	119	2

## Results

### Overall Findings

#### Influence of Game Modes on Total Playing Time

The analysis revealed that the average total playing time per game mode (in mm:ss) varied slightly among different game modes. On average, the creative mode (mean 16:39, SD 6:45) was the longest, followed by the competitive mode (mean 16:01, SD 5:46), and the cooperative mode (mean 14:40, SD 6:32). The playing times did not differ significantly among game modes as shown by the Friedman test (*P*=.67). Playing time for each mode was restricted by the study instructions to a maximum of 20 minutes.

#### Influence of Game Modes on Total Time of Social Interactions

**Figure 4:**
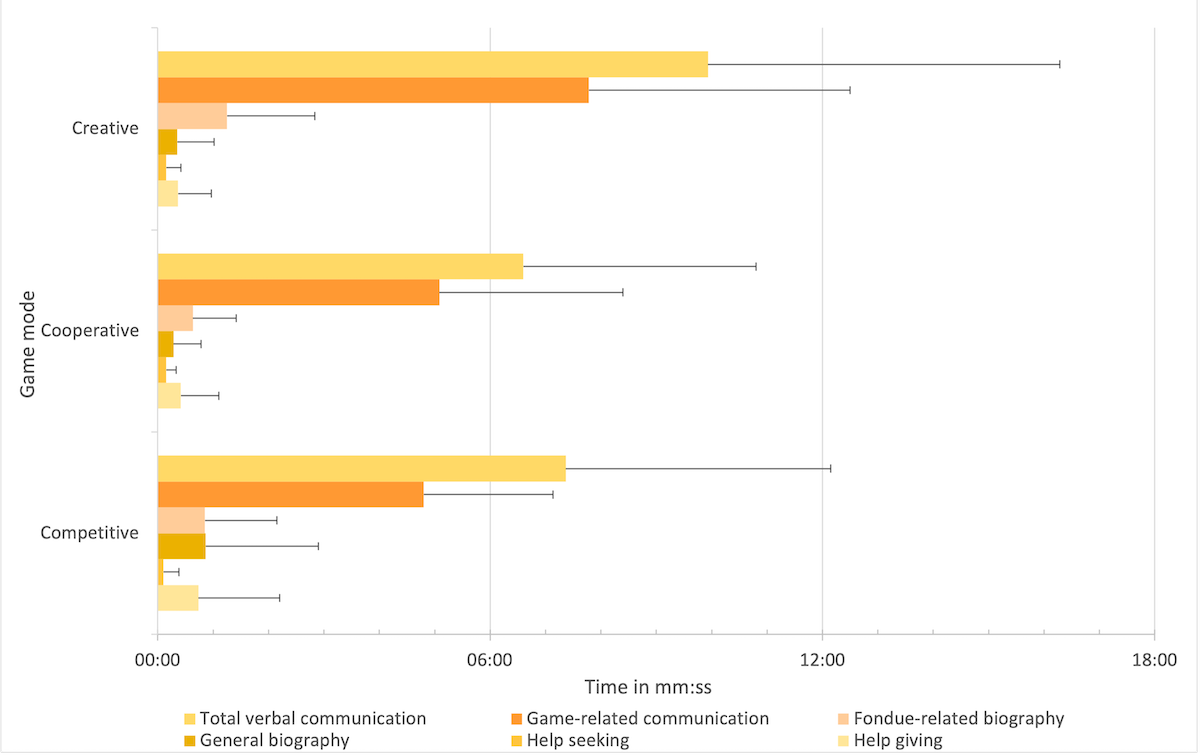
Verbal communication (absolute values).

**Figure 5:**
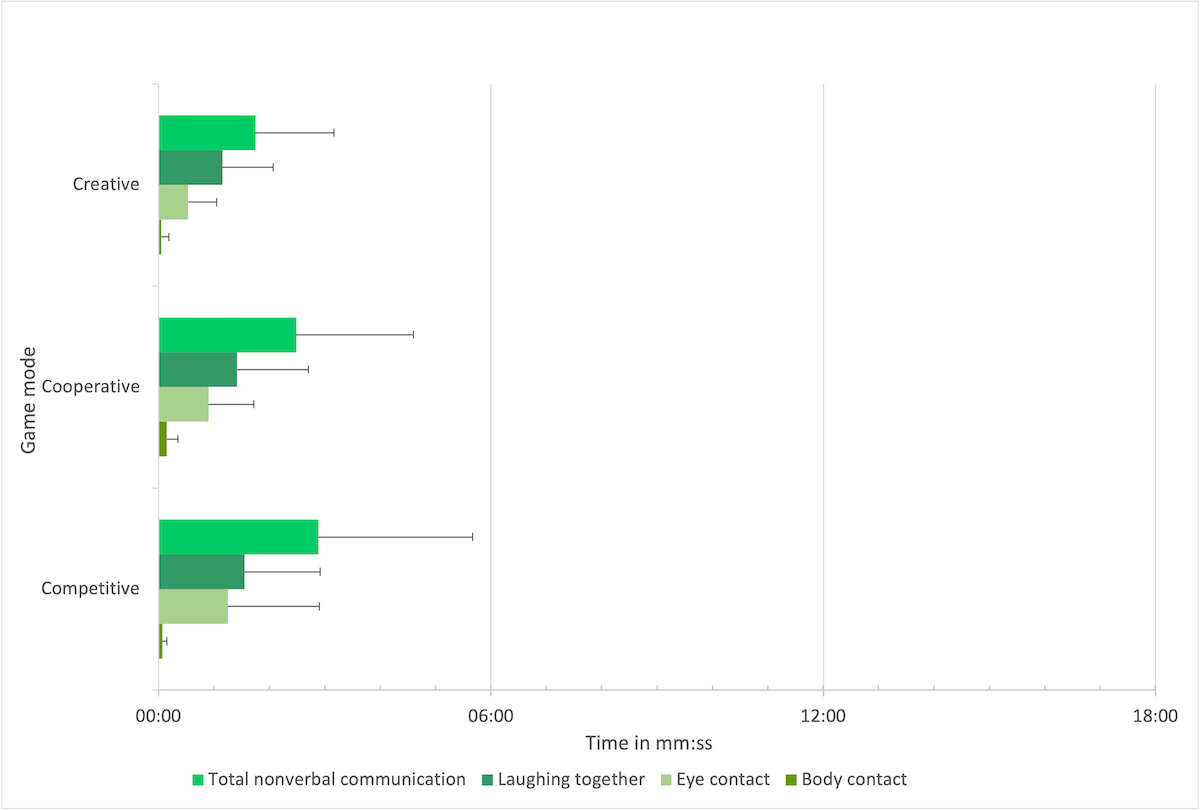
Nonverbal communication (absolute values).

**Figure 6:**
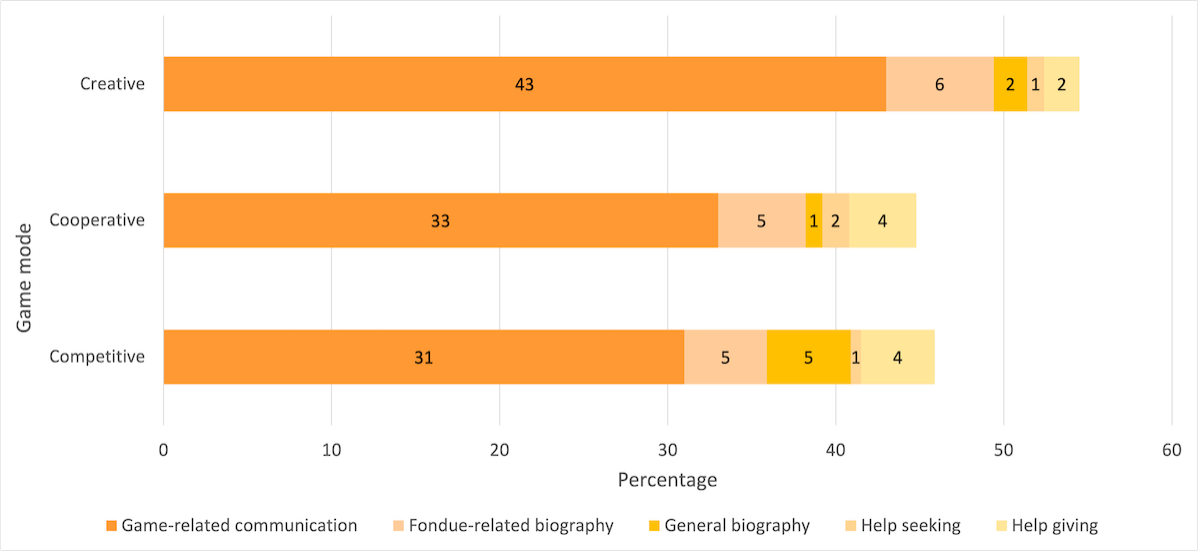
Verbal communication (relative values).

**Figure 7:**
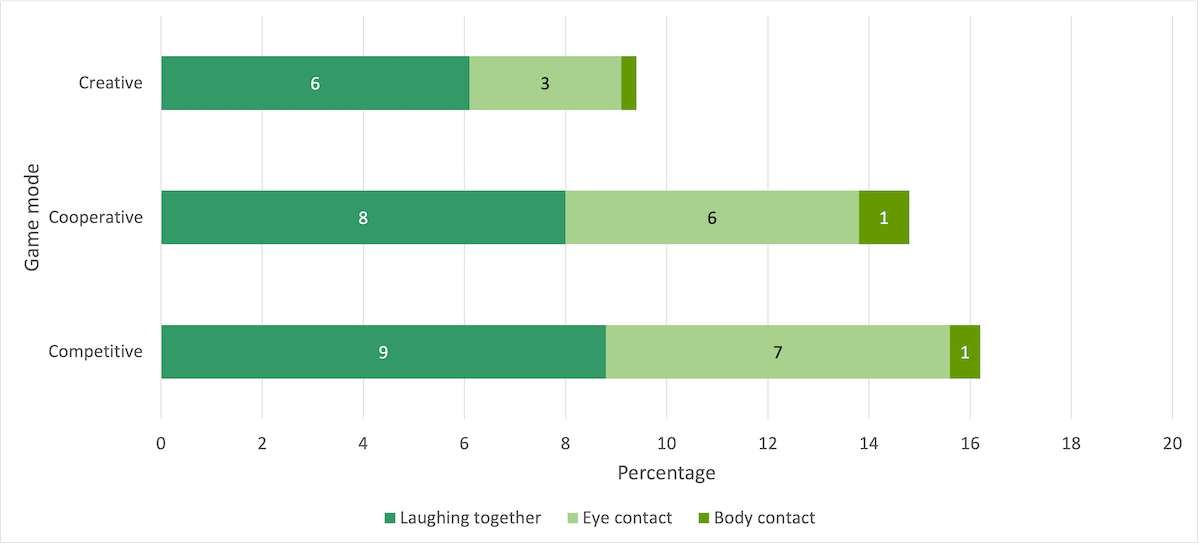
Nonverbal communication (relative values).

The results revealed that for approximately half of the total playing time (in hh:mm:ss), social interactions were observed (3:51:47 with 7570 coded social interactions). It is important to note that different categories of social interaction occurred simultaneously, and the addition of these categories resulted in an *artificially* prolonged total duration (5:10:34, 7570 coded social interactions). Table 3 summarizes the absolute and relative values of verbal and nonverbal social interactions (along with the respective subcategories) for the different game modes. Figures 4-7 show a graphical depiction of these results.

**Table 3:** Absolute and relative values of social interaction in the different game modes.

Social interaction category				Game mode							Hypothesis	
				Creative		Cooperative		Competitive				
**Verbal, mean (SD)**												H1^a^
	**Game-related communication**											
		Absolute values (mm:ss)	7:47 (4:43)		5:05 (3:19)		4:48 (2:20)		Hypothesis confirmed^b^			
		Relative values (% of total time)	43 (18.7)		33 (15.8)		31.2 (16.4)		Hypothesis confirmed^c^			
	**Fondue-related biography**											
		Absolute values (mm:ss)	1:15 (1:35)		0:38 (0:47)		0:51 (1:18)		Hypothesis partly confirmed^d^			
		Relative values (% of total time)	6.4 (7.3)		5.2 (7.8)		4.9 (6.6)		—^e^			
	**General biography**										—	
		Absolute values (mm:ss)	0:21 (0:40)		0:17 (0:30)		0:52 (2:02)					
		Relative values (% of total time)	1.8 (3.3)		1.4 (2.3)		4.9 (9.9)					
	**Help seeking**											
		Absolute values (mm:ss)	0:09 (0:16)		0:09 (0:11)		0:06 (0:17)		—			
		Relative values (% of total time)	1 (1.6)		1.6 (2.3)		0.6 (1.4)		Hypothesis partly confirmed^f^			
	**Help giving**										—	
		Absolute values (mm:ss)	0:22 (0:36)		0:25 (0:41)		0:44 (1:28)					
		Relative values (% of total time)	2.1 (3.4)		4 (6.2)		4.4 (7.3)					
	**Total verbal communication**											
		Absolute values (mm:ss)	9:56 (6:21)		6:36 (4:12)		7:22 (4:47)		Hypothesis partly confirmed^d^			
		Relative values (% of total time)	54 (25.9)		45 (20.4)		46 (25.3)		—			
**Nonverbal, mean (SD)**												H2^g^
	**Laughing together**											
		Absolute values (mm:ss)	1:09 (0:55)		1:25 (1:17)		1:33 (1:22)		—			
		Relative values (% of total time)	6.1 (4.1)		8 (5.5)		8.8 (6.3)		Hypothesis confirmed^c^			
	**Eye contact**										—	
		Absolute values (mm:ss)	0:32 (0:31)		0:54 (0:49)		1:15 (1:39)					
		Relative values (% of total time)	3 (2.4)		5.8 (4.3)		6.8 (7.8)					
	**Body contact**										—	
		Absolute values (mm:ss)	0:03 (0:08)		0:09 (0:12)		0:04 (0:05)					
		Relative values (% of total time)	0.3 (0.7)		1 (1.2)		0.6 (0.8)					
	**Total nonverbal communication**											
		Absolute values (mm:ss)	1:45 (1:25)		2:29 (2:07)		2:53 (2:47)		—			
		Relative values (% of total time)	9.4 (6.2)		14.8 (9.3)		16.2 (12.5)		Hypothesis confirmed^b^			
**Total social interaction**												—
	Absolute values (mm:ss)		11:41 (7:11)		9:06 (5:45)		10:16 (6:44)					
	Relative values (% of total time)		63.7 (28)		59.9 (23)		62.2 (31.1)					

^a^H1: hypothesis 1.

^b^Significance, *P*=.01.

^c^Significance, *P*=.02.

^d^Marginal significance, *P*=.06.

^e^Hypothesis not confirmed.

^f^Significance, *P*=.04.

^g^H2: hypothesis 2.

As seen in Table 3, on a descriptive level, the *absolute total values* of social interaction were the longest in the creative mode, followed by the competitive and cooperative modes. However, the analysis of variance for repeated measurements showed no significant differences among the game modes (*P*=.29). The same holds true for the *relative values* (Table 3), that is, percentage of time with observed social interaction in relation to the total playing time (*P*=.78).

### Simultaneous Occurrence of Verbal and Nonverbal Codes

As verbal and nonverbal categories tend to occur simultaneously, overlapping frequencies between verbal and nonverbal codes are presented in Table 2. During game-related communication, players often laugh together and look at each other in their eyes. However, during biographic communication (ie, fondue-related and general biography), players rarely had eye contact or laughed together. Regardless of the content of verbal communication, body contact rarely occurred.

### Hypothesis 1: Verbal Communication

#### Influence of Game Modes on Total Verbal Communication

On a descriptive level, the *absolute total values* of verbal communication were higher in the creative mode, followed by the competitive and cooperative modes (Table 3). Analysis of variance for repeated measures yielded marginal significance (*F*_2,18_=3.37; *P*=.06; partial η^2^=0.272; N=10). The effect size *f,* according to Cohen (1988), was 0.611, which corresponds to a strong effect. Bonferroni-corrected pairwise comparisons revealed that there was significantly more verbal communication (*P*=.04) in the creative mode (mean 9:56, SD 6:21) than in the cooperative mode (mean 6:36, SD 4:12). For the *relative values,* the analyses did not yield statistical significance (*P*=.11).

#### Influence of Game Modes on Game-Related Communication

As seen in Table 3, on a descriptive level, the *absolute values* of game-related verbal communication were higher in the creative mode, followed by the cooperative and competitive modes. An analysis of variance for repeated measurements showed that the *absolute values* of game-related communication differed significantly among the game modes (*F*_2,18_=6.36; *P*=.01; partial η^2^=0.414; N=10), with a strong effect (Cohen *f*=0.841). Bonferroni-corrected pairwise comparisons showed that there was significantly more game-related communication (*P*=.01) in creative mode (mean 7:47, SD 4:43) than in cooperative mode (mean 5:05; SD 3:19) and marginally significantly more game-related communication in cooperative mode (*P*=.09) than in competitive mode (mean 4:48, SD 2:20).

Analysis of variance for repeated measurements also showed significant differences among game modes (*F*_2,18_=4.85; *P*=.02; partial η^2^=0.350; N=10), with a strong effect (Cohen *f*=0.734) for the *relative values* of game-related communication. Bonferroni-corrected pairwise comparisons revealed that in the creative mode (mean 43%, SD 18.7%), the percentage of game-related communication was significantly higher (*P*=.01) than in the cooperative mode (mean 32.9%, SD 15.8%).

#### Influence of Game Modes on Fondue-Related Biography Talk

As seen in Table 3, on a descriptive level, the *absolute values* of fondue-related biography communication were higher in the creative mode, followed by the competitive and cooperative modes. According to relative values, values were higher in the creative mode than in the cooperative mode, followed by the competitive mode. The Friedman test showed marginally significant differences among the game modes for the *absolute values* (*χ*^2^_2_=5.56; *P*=.06; N=10), but not for the *relative values* (*P*=.37; Table 3). For the absolute values, post hoc tests (Dunn-Bonferroni tests) revealed significantly more verbal communication regarding fondue-related biography (*z*=2.24; *P*=.03) in the creative mode (mean 1:15, SD 1:35) than in the cooperative mode (mean 0:38, SD 0:47) with a strong effect (*r*=0.707).

#### Influence of Game Modes on General Biography Talk

As seen in Table 3, on a descriptive level, the *absolute values* of general biography were higher in the competitive mode, followed by the creative and cooperative modes. No significant differences were found among the game modes (*P*=.91). This also holds true for *relative values* (*P*=.91).

#### Influence of Game Modes on Help Seeking and Help Giving

Table 3 shows on a descriptive level the *absolute values* of help seeking and help giving. No significant differences were found between the game modes for help seeking (*P*=.28) and help giving (*P*=.47). For the relative values, a Friedman test showed a significant difference among the game modes (χ^2^_2_=6.44; *P*=.04; N=10) for help seeking. Subsequent post hoc tests (Dunn-Bonferroni tests) revealed that help seeking was significantly higher (*z*=2.24; *P*=.03) in the cooperative mode (mean 1.6%, SD 2.3%) than in the competitive mode (mean 0.6%, SD 1.4%) with a strong effect (*r*=0.707). Concerning the absolute and relative values for the category help giving, no significant differences were found among the game modes.

In sum, we expected the creative game mode to stimulate the highest amount of verbal social interaction, because players are given a high degree of freedom, which could stimulate verbal interaction. Despite somewhat controversial findings regarding the competitive and cooperative modes, we assumed in this study that a cooperative mode would stimulate more verbal social interaction than the competitive mode based on related research (H1). The hypothesis could partly be confirmed: for total verbal communication and the subcategories game-related and fondue-related communication, where data indeed show higher values for the creative mode, indicating more time spent on verbal communication here than in the other modes, partly with strong effects. With regard to the effects of cooperative and competitive modes, the picture is less clear. The same is true for the other subcategories (general biography talk, help seeking, and help giving). Considering the rationale behind the assumptions of H1, this pattern of results is interesting and can be explained so that in creative mode, more verbal communication occurs, because players are given a high degree of freedom to exchange and develop new ideas. However, as the data show, this could only hold true for *some* subcategories of communication, namely those that directly relate to playing the game itself (game-related and fondue-related biography). On a more general level, game modes may influence verbal communication in different ways, but only for specific content.

### Hypothesis 2: Nonverbal Communication

#### Influence of Game Modes on Total Nonverbal Communication

The mean *absolute values* of nonverbal communication, as shown in Table 3, were highest in the competitive mode, followed by the cooperative and creative modes. An analysis of variance for repeated measurements (Greenhouse–Geiser correction was applied) indicated marginally significant differences (*P*=.09), but the differences did not reach significance in the subsequent post hoc tests (*P*=.14, *P*=.26, and *P*=.84).

A Friedman test for the *relative* values showed a significant difference among the game modes (*χ*^2^_2_=9.80; *P*=.01; N=10). Following post hoc tests (Dunn-Bonferroni tests) revealed that the nonverbal communication was significantly higher (*z*=−2.91; *P*=.004) in the competitive mode (mean 16.2%, SD 12.5%) than in the creative mode (mean 9.4%, SD 6.2%) with a strong effect (*r*=0.919), and significantly higher (*z*=−2.46; *P*=.01) in the cooperative mode (mean 14.8%, SD 9.3%) than in the creative mode with a strong effect (*r*=0.778).

In the following sections, the results will be reported for different categories of nonverbal communication.

#### Influence of Game Modes on Laughing Together

The mean *absolute values* of laughing together, as shown in Table 3, were highest in the competitive mode, followed by the cooperative and creative modes. An analysis of variance for repeated measurements with the absolute values for laughing together showed a marginally significant difference among game modes (*P*=.08) but this difference was not significant in the subsequent post hoc tests (*P*=.27, *P*=.26, and *P*=.99). Regarding the *relative values*, an analysis of variance for repeated measurements showed significant differences among the game modes (*F*_2,18_=5.18; *P*=.02; partial η^2^=0.365; N=10), with a strong effect (Cohen *f*=0.758). Bonferroni-corrected pairwise comparisons revealed that there was more laughing together in the competitive mode (mean 8.8%, SD 6.3%) than in the creative mode (mean 6.1%, SD 4.1%; *P*=.047) and marginally significantly more laughing together (*P*=.07) in the cooperative mode (mean 8%, SD 5.5%) than in the creative mode.

#### Influence of Game Modes on Eye Contact

There was no significant difference between the game modes for either the absolute (*P*=.21) or the relative values of eye contact (*P*=.12).

#### Influence of Game Modes on Body Contact

There was no significant difference between the game modes for either the absolute (*P*=.63) or the relative values of body contact (*P*=.41).

In sum, regarding nonverbal social interaction, it was expected that there would be differences among game modes (H2), but expectations remained on an explorative level, owing to a lack of a theoretical or empirical research basis for directional assumptions. The results reveal that the total time spent with nonverbal communication and laughing together was highest in the competitive mode and lowest in the creative mode, with significant differences. No significant differences were found between the eye and body contact subcategories.

## Discussion

### Principal Findings

In the study presented here, the influence of 3 different game modes of the multiplayer video game *Myosotis FoodPlanet* on the social interactions between the older players living in a retirement home and their younger coplayers was investigated. It was expected that different game modes would influence verbal and nonverbal communication among players in different ways (H1 and H2)*.*

First, overall (across all modes), it was demonstrated that the game could successfully be applied and played as intended in the field situation (retirement home) by the residents and their younger coplayers (care professionals). All 3 game modes could *successfully* stimulate positive social interactions, such as talking and laughing together between the older adults and their younger coplayers (albeit in different ways, see below). Simultaneous occurrences (Table 2) of game-related verbal communication and the 2 nonverbal categories of laughing together and eye contact were found. Regardless of the game mode, players tended to stop playing and listen carefully when the other player told a story from the past. Overall, these study results are in line with previous research showing that game-mediated play paves the way for positive social interactions [13,14] and can facilitate communication among players belonging to different age groups or generations [9,10,12]. Thus, important practical goals of the study were met, as it was specifically designed as an intergenerational digital game for use in retirement homes. The game was designed to be fun and entertaining for different generations to support mutual empathy and active listening, and it obviously worked.

Second, partly confirming our assumptions about *the differential* effects of *different* game modes on social interaction, some significant differences were found. To highlight the most important outcomes, it is noted that the creative game mode was significantly more supportive than the other modes with respect to the subcategories of verbal communication, game-related communication, and fondue-related biography talk but not for general biography talk or help giving. In other words, the creative game mode of *Myosotis FoodPlanet* could—better than other game modes—foster social interactions between older adults and their younger coplayers regarding talking about the game and their joint activities during playing. The older adults seemingly related their memories from the past to the present game content (ie, remembering past fondue-events). Furthermore, our results revealed significantly more help seeking communication in the cooperative mode than in the competitive mode. Taken together, these findings support H1. However, with regard to the cooperative and competitive modes, the picture remains fuzzy. The results were inconsistent, mirroring the situation in previous research, where results from empirical studies comparing different modes in relation to social interaction are inconsistent, as described above (in the introduction section on game design decisions and their possible influences on social interaction). Finally, the competitive mode was significantly better than the other modes in promoting the subcategory of laughing together in nonverbal social interaction, whereas no effects were found for eye or body contact. Therefore, H2 was only partly confirmed. This result is in line with related research showing a positive relationship between social and competition motives [39,40]. This seemingly contradicts previous results showing that older adults (in contrast to younger players) would find competition in playing a minor motivator [16,18,22,27]. This can be explained by the unthreatening but still exciting and humorous character of the specific game *Myosotis FoodPlanet* used here.

In summary, the results show how design decisions concerning the choice of game mode can be an important factor in shaping the social interactions of players of different age groups while playing together. In a scientific sense, these original results add to the research gap identified in research on intergenerational games [23], because they provide comparative evidence that can be explained by theory. The creative mode included a high level of freedom for the players and practically demanded communication during the game to *prepare* the players’ favorite fondue. It stimulated negotiations about what ingredients to use, resulting in more game-related communication, compared with the other modes. In addition, the creative mode provided an environment in which the players were not under the pressure of wanting to win. Hence, they could talk freely while playing the game, which in turn could have stimulated the cued recall of associated individual memories concerning fondue-related biographic communication.

### Strengths and Limitations

This study has its strengths and limitations. An important strength of this study pertains to the study setting. In comparison to previous studies, which were often conducted in a laboratory situation and therefore might lack ecological validity, our study was conceptualized as a field trial. Thus, we could examine the participants in their natural environment, which is important when looking at aspects such as social interaction and drawing real-world conclusions [57]. However, there are limitations to this approach. In this section, we address these limitations and justify the value of the study despite these limitations.

The small number of participants involved was a serious limitation. This was owing to practical limitations on the side of the retirement homes (eg, their willingness, trust, and available resources to undergo the effort of participating in this field research with researchers coming to their place, bringing in video games for the residents, and placing video cameras to perform recordings). We justify the study despite this limitation by the amount of field data gathered despite the low number of participants. We recorded 30 game sequences, resulting in approximately 8 hours of video material (7 hours 53 minutes 25 seconds). Social interactions were observed, comprising a volume of almost 4 hours of video (3:51:47) with 7570 coded social interactions in the different categories, coded thoroughly according to well-established methods in experimental psychology. Our fine-grained videotaping and coding procedure allowed us to analyze both verbal and especially, nonverbal communication, from which we gained crucial insights into the differences among the game modes. Hence, an important further strength of this study arises from this systematic effort: not only verbal communication but also nonverbal interactions, such as laughing together or eye contact are available and produced interesting new results in this area of research. However, most importantly, significant results were obtained despite the small number of participants (N), and sometimes with effect sizes indicating strong effects, such as the results on the influence of game modes on game-related and fondue-related biography talk, and on total nonverbal communication (see the *Results* section)*.*

We must also note that the younger coplayers in this study were care professionals. Although this fills a gap in research on intergenerational games by looking *further than only grandparents–grandchildren interactions* [23], this may have compromised our study and demands additional caution in interpretation and generalization. Such professionals are trained to read the skill levels of the older adults and adapt their own pace accordingly. Furthermore, older residents may have viewed them as their *caregivers*, in addition to viewing them as *members of the younger generation*. Thus, it remains open at this point how nontrained younger coplayers or family members would behave in such gaming situations and how social interactions would differ from the situation studied here. For instance, it may be that some study outcomes would look different; with family members as younger coplayers, there might be more (joint) general biography-related memories to discuss. The players may invest more time in general biography talk with implications for the effects of the 3 game modes on this subcategory of social interaction. In addition, the patterns of help seeking and help giving types of communication would likely produce different results. The same might be true for body contact. This limitation characterizes our study outcomes as *intergenerational* only in terms of age (younger vs older) but does not necessarily generalize to other characterizations such as family links (eg, grandparents and grandchildren) or organizational membership (eg, juniors and seniors) or other [24]. This may be the subject of future studies.

The game used in this study is a serious multiplayer game called *Myosotis FoodPlanet*, which was designed specifically as a serious intergenerational game for practical use in retirement homes. It was also used for local game events with older adults in different areas of Switzerland. Using this real game supports the ecological validity of the study but somewhat compromises internal validity, as control over all elements and features in the game was not possible. During the game design process, we could not control for all possible confounds across game modes; for example, the creative mode includes some additional elements, such as additional effects and recipes with humorous names. This must be discussed in terms of alternative explanations for the findings showing more verbal communication in the creative mode than in the other modes (confirming H1): Could it be that more talking was stimulated by a more humorous game mode? When viewed together with the results on nonverbal communication, this alternative seems rather unlikely, as significantly more time of *laughing together* was found in the competitive mode than in the creative mode. This suggests that humor was not limited to the creative game mode but a characteristic of the game *Myosotis FoodPlanet* in all modes investigated here. Therefore, we understand our study results still as confirming H1. Nevertheless, this interpretation must remain cautious and initial. Further systematic experimental studies are needed to deepen scientific knowledge on this specific topic.

Associated with the previous information, the game addresses a specific theme (Swiss fondue cooking), represents a specific genre (casual), and has a specific target (intergenerational game playing in retirement homes to improve social interactions). Generalization of the study results to other games and situations is not easily possible. Thus, comparisons among different games (genres, mechanics, and themes) and coplayer’s profiles in systematic experimental research could be interesting in future research.

Nevertheless, it is a strength of this study in comparison with previous work that it examines the creative game mode with this specific game in a field trial and that it systematically compares the effects of the 3 game modes on social interactions in a natural setting. Previous studies have primarily addressed the influence of either cooperative or competitive play on social interaction [33,38], whereas a direct comparison of cooperative, competitive, and creative game modes regarding their similarities and differences in promoting different types of social interaction has been lacking so far.

### Conclusions

What does the study reveal for the community at the end? In sum, this study highlights the importance of game mode when designing serious multiplayer games for intergenerational game playing and can inform game designers on *how* to design games to stimulate specific types of intergenerational social interaction. Moreover, the results have implications for game-playing situations, such as the one investigated here in a retirement home, when the aim is to promote intergenerational social interaction between care professionals and residents (younger and older). Depending on the type of social interaction (eg, verbal or nonverbal communication) that is intended, a specific game mode or specific elements may be more appropriate than others. When an increase in verbal communication is desired and when older adults should be motivated to talk with younger (care) persons, a creative game mode with no distinct goal and no right or wrong pathway should be considered. On the contrary, when an increase in laughing together is intended (eg, to provide for a positive mood and atmosphere), one might recommend a competitive game mode with an unthreatening, though arousing and stimulating character. These conclusions remain tentative owing to the study limitations and limited research in this area. We hope that our study will stimulate future research.

## Appendix

Multimedia Appendix 1 Game design of the Myosotis Fondue game.

Multimedia Appendix 2 CONSORT-eHEALTH checklist (V 1.6.1).
